# Association among chronic kidney disease, airflow limitation, and mortality in a community-based population: The Yamagata (Takahata) study

**DOI:** 10.1038/s41598-020-62540-8

**Published:** 2020-03-27

**Authors:** Natsuko Suzuki, Eri Matsuki, Akira Araumi, Sakiko Ashitomi, Sayumi Watanabe, Kosuke Kudo, Kazunobu Ichikawa, Sumito Inoue, Masafumi Watanabe, Yoshiyuki Ueno, Kenichi Ishizawa, Takamasa Kayama, Tsuneo Konta

**Affiliations:** 1 Department of Cardiology, Pulmonology, and Nephrology, Yamagata University, Yamagata, Japan; 2 Institute for Promotion of Medical Science Research, Yamagata University, Yamagata, Japan; 3 Department of Public Health and Hygiene, Yamagata University, Yamagata, Japan

**Keywords:** Kidney diseases, Epidemiology

## Abstract

Chronic kidney disease (CKD) and chronic obstructive pulmonary disease (COPD) are known risk factors for mortality. In this study, we examined the overlap of CKD and airflow limitation (AFL) that characterises COPD and its effect on 10-year mortality in a community-based population. This study included 1,233 health check-up participants (mean age, 63.7 years; 46.7% men). We defined serum creatinine-based CKD (CKDcr) and serum cystatin C-based CKD (CKDcys) as glomerular filtration rate <60 mL/min/1.73 m^2^, estimated using serum creatinine or cystatin C, and/or dipstick proteinuria ≥1+. AFL was defined as forced expiratory volume in 1 s to forced vital capacity ratio <70% on spirometry. Compared with subjects without AFL, those with AFL showed a significantly higher prevalence of CKDcys but not of CKDcr. Cox proportional hazard analysis adjusted for confounders showed that the hazard ratio (95% confidence interval) for all-cause mortality was 1.45 (0.77–2.63) in subjects with CKDcys alone, 1.29 (0.60–2.54) in those with AFL alone, and 2.94 (1.33–6.12) in those with both CKDcys and AFL, with subjects without both AFL and CKD as the reference. This study showed that AFL and CKDcys are strongly associated and that their overlap is a significant risk factor for mortality in community-based populations.

## Introduction

As population ageing progresses in developed countries, including Japan, the numbers of patients with chronic kidney disease (CKD) and chronic pulmonary obstructive disease (COPD) increase. CKD is defined as ‘a pathological condition in which renal impairment such as proteinuria or glomerular filtration rate (GFR) < 60 mL/min/1.73 m^2^ persists for>3 months’^1^. The prevalence of CKD is >10% among the worldwide population^1^, and CKD has the second highest mortality rate increase worldwide, after human immunodeficiency virus infection and acquired immunodeficiency syndrome^2^. COPD is defined as ‘a common, preventable, and treatable disease that is characterised by persistent respiratory symptoms and airflow limitation (AFL) due to airway and/or alveolar abnormalities, usually caused by significant exposure to noxious particles or gases’^3^. It is estimated that there are currently 384 million patients with COPD worldwide^4^, and COPD is expected to become the third leading cause of global death by 2030^5^. CKD and COPD are now common diseases, and their overlap is sometimes observed among the general population. A previous cross-sectional study reported that renal insufficiency is independently associated with COPD in the general population^6^. However, there has been no report regarding the effect of the combination of CKD and COPD on life prognosis.

In general practice, renal function is evaluated based on serum creatinine (Cr) level and GFR is estimated using the Cr-based formula (eGFRcr). However, for patients with lean muscle mass who have reduced serum Cr production, eGFRcr might overestimate renal function, resulting in failure to detect the presence of CKD. In such cases, it is recommended to use other markers of renal function, such as serum cystatin C (CysC) and CysC-based eGFR (eGFRcys), which are less susceptible to lean muscle mass than serum Cr and eGFRcr^7^. Because patients with COPD are often lean, it may be more appropriate to use CysC rather than Cr for detecting CKD in these patients. In a Japanese hospital-based study, eGFRcys was significantly lower than eGFRcr in patients with COPD^8^. Our previous study revealed that eGFRcys showed a stronger association with mortality than eGFRcr in a community-based population^9^. However, the association between CKD evaluated using eGFRcr or eGFRcys and COPD among community-based populations is unknown.

Therefore, in this cohort study, we examined the association between CKD and AFL that characterises COPD and the effect of their overlap on mortality in a community-based population using serum Cr and CysC as markers of renal function.

## Results

**Figure 1:**
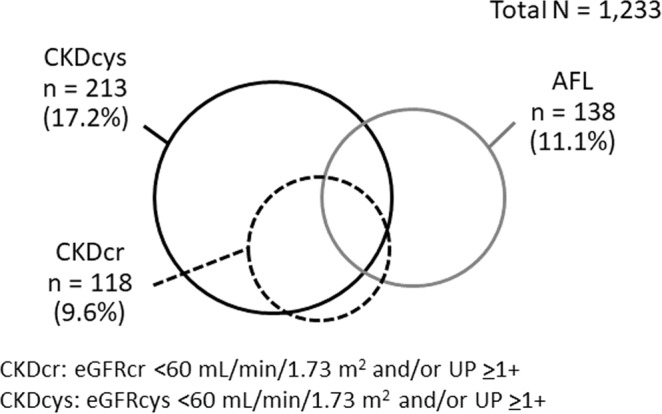
Distribution of CKDcr, CKDcys, and AFL among the study subjects.

At baseline, among all subjects, the prevalence of eGFRcr <60 mL/min/1.73 m^2^, eGFRcys <60 mL/min/1.73 m^2^, and dipstick proteinuria ≥1+ was 6.6%, 14.8%, and 3.8%, respectively. The prevalence of CKDcr (eGFRcr <60 mL/min/1.73 m^2^ and/or dipstick proteinuria ≥1+), CKDcys (eGFRcys <60 mL/min/1.73 m^2^ and/or dipstick proteinuria ≥1+), and AFL was 9.6%, 17.2%, and 11.1%, respectively (Fig. 1). The baseline characteristics of the study subjects classified using the combination of CKDcys and AFL are shown in Table 1. The average age and prevalence of men, smoking, alcohol consumption, and diabetes were higher and the prevalence of hypertension and hyperlipidaemia was lower in subjects with both CKDcys and AFL than in those without CKDcys and AFL. A similar tendency was observed in subjects classified using the combination of CKDcr and AFL.

**Table 1:** Baseline characteristics of the study subjects.

	Total N = 1,233	CKDcys/AFL				P-value
		(−)/(−) n = 918	(+)/(−) n = 177	(−)/(+) n = 102	(+)/(+) n = 36	
Age (years)	63.7 ± 9.8	61.8 ± 9.5	69.8 ± 8.9	67.0 ± 6.9	71.9 ± 6.7	<0.01
Male sex (%)	569 (46.7%)	388 (42.2%)	80 (45.2%)	70 (68.6%)	31 (86.1%)	<0.01
Smoking status (%)	164 (13.3%)	107 (11.7%)	22 (12.4%)	26 (25.5%)	9 (25.0%)	<0.01
Alcohol consumption (%)	516 (41.8%)	376 (41.0%)	53 (29.9%)	65 (63.7%)	22 (61.1%)	<0.01
Body mass index (kg/m^2^)	23.5 ± 3.2	23.5 ± 3.2	24.3 ± 3.5	22.7 ± 3.2	23.2 ± 3.1	0.01
Serum Cr (mg/dL)	0.67 ± 0.16	0.65 ± 0.13	0.77 ± 0.23	0.67 ± 0.13	0.83 ± 0.16	<0.01
Serum cystatin C (mg/L)	0.95 ± 0.19	0.89 ± 0.12	1.21 ± 0.24	0.93 ± 0.11	1.24 ± 0.15	<0.01
Hypertension (%)	532 (43.2%)	381 (41.5%)	98 (55.4%)	45 (44.1%)	8 (22.2%)	<0.01
Diabetes (%)	67 (5.8%)	43 (4.9%)	13 (7.9%)	3 (3.2%)	8 (24.2%)	<0.01
Hyperlipidaemia (%)	378 (30.7%)	292 (31.8%)	56 (31.6%)	27 (26.5%)	3 (8.3%)	0.02

CKDcys, cystatin C-based chronic kidney disease; AFL, airflow limitation; Cr, creatinine.

**Figure 2:**
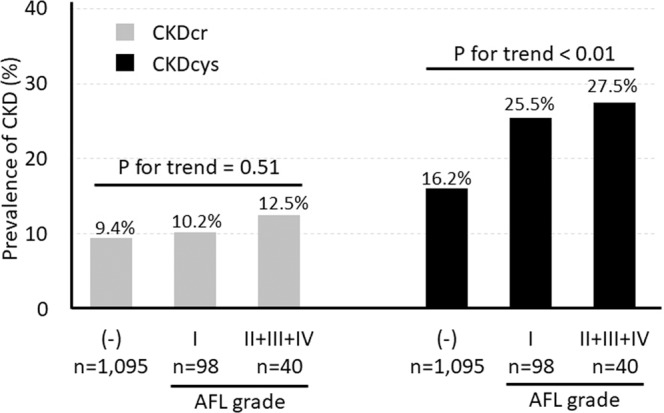
Prevalence of CKD according to AFL grade.

In the cross-sectional analysis, we first examined the prevalence of CKD in subjects with and without AFL. The results showed that the prevalence of CKDcys was significantly higher in subjects with AFL than in those without (26.1% vs 16.2%, P < 0.01). In contrast, the prevalence of CKDcr was not different between subjects with and without AFL (10.9% vs 9.4%, P = 0.58). We further examined the association between CKD and the AFL grade and found that the prevalence of CKDcys, but not CKDcr, increased as the AFL grade increased (P for trend <0.01) (Fig. 2). We also examined the prevalence of AFL according to the presence and absence of CKD. The results showed that the prevalence of AFL was significantly higher in subjects with CKDcys than in those without (16.9% vs 10.0%, P < 0.01), but not in subjects with or without CKDcr (12.7% vs 11.0%, P = 0.58). These results indicate that the association of AFL with CKDcys seems to be stronger than their association with CKDcr.

**Figure 3:**
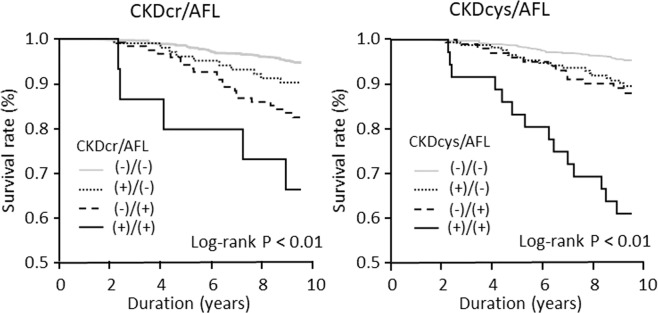
Kaplan-Meier curves of the combination of CKD and AFL versus mortality.

During the 10-year follow-up from 2004 to 2013, 86 subjects died. In the longitudinal analysis, we first performed a c-statistics analysis to evaluate the goodness of fit in a logistic regression model for mortality using eGFRcr or eGFRcys, and found that the value of c-statistics was significantly higher in Cys-based eGFR (0.67, 95% confidence interval [CI] 0.61–0.73) than Cr-based eGFR (0.57, 95% CI 0.51–0.64) (P < 0.01). In Cox proportional hazard model to predict mortality the P value for interaction was 0.834 for Cr-based CKD and AFL, and 0.254 for Cys-based CKD and AFL. Then we performed Kaplan-Meier survival analysis to examine the association between CKD and AFL overlap and mortality. The results showed that the survival rate was the lowest in subjects with both CKDcr or CKDcys and AFL (log-rank P < 0.01) (Fig. 3). Thereafter, to examine the independent association between the combination of AFL and CKDcys on all-cause mortality, we performed Cox proportional hazard analysis adjusted for possible confounders. The results showed that the adjusted hazard ratio (95% CI) was 1.45 (95% CI 0.77–2.63) in subjects with CKDcys alone, 1.29 (95% CI 0.60–2.54) in those with AFL alone, and 2.94 (95% CI 1.33–6.12) in those with both CKDcys and AFL, with subjects without both AFL and CKD as the reference. In contrast, when renal function was evaluated using Cr, the adjusted hazard ratios showed a similar tendency, although they did not reach statistical significance in all categories: 1.47 (95% CI 0.69–2.84) in subjects with CKDcr alone, 1.76 (95% CI 0.97–3.06) in subjects with AFL alone, and 2.42 (95% CI 0.70–6.33) in subjects with both CKDcr and AFL, with subjects without both AFL and CKD as the reference (Table 2).

**Table 2:** Association between CKD and AFL overlap and mortality.

CKDcr/AFL	Number of mortality/total subjects	Observational period (person-year)	Incidence rate (/1,000 person-year)	Unadjusted			Adjusted*		
				HR	95% CI	P-value	HR	95% CI	P-value
(−)/(−)	50/992	9085.8	5.5	reference			reference		
(+)/(−)	10/103	929.6	10.8	1.97	0.94–3.72	0.07	1.47	0.69–2.84	0.30
(−)/(+)	21/123	1079.4	19.5	3.59	2.12–5.90	<0.01	1.76	0.97–3.06	0.06
(+)/(+)	5/15	118.7	42.1	8.05	2.80–18.3	<0.01	2.42	0.70–6.33	0.14
CKDcys/AFL				HR	95% CI	P-value	HR	95% CI	P-value
(−)/(−)	42/918	8420.6	5.0	reference			reference		
(+)/(−)	18/177	1594.8	11.3	2.36	1.33–4.21	<0.01	1.45	0.77–2.63	0.24
(−)/(+)	12/102	913.5	13.1	2.78	1.41–5.47	<0.01	1.29	0.60–2.54	0.49
(+)/(+)	14/36	284.7	49.2	13.3	6.34–27.8	<0.01	2.94	1.33–6.12	<0.01

*Adjusted for age, sex, smoking status, alcohol consumption, body mass index, hypertension, diabetes, and hyperlipidaemia.

HR, hazard ratio; CI, confidence interval; CKD, chronic kidney disease; AFL, airflow limitation; CKDcr, creatinine-based chronic kidney disease; CKDcys, cystatin C-based chronic kidney disease.

## Discussion

In this cohort study, we showed for the first time that CKDcys and AFL are likely to overlap in community-based populations and that the overlap of AFL and CKDcys additively increases the risk of all-cause mortality. In addition, serum cystatin-based eGFR might be a better index for evaluating the risk of mortality than serum Cr-based eGFR in subjects with AFL.

In clinical practice, renal function is usually evaluated using serum Cr levels. However, in subjects with low muscle mass, which translates to reduced production of Cr from muscles, such as subjects with amputated limbs or those in a long-term bedridden state, the serum Cr levels often stay within the normal range even in the presence of decreased renal function. The same problem can be observed in patients with COPD, which is related to muscle loss and leanness^9^, and sarcopenia progresses with age and the extent of airflow obstruction^10^. In contrast, CysC was reported to be more sensitive than Cr in detecting mildly impaired renal function and was not affected by the muscle mass volume^11^. A cross-sectional analysis revealed that eGFRcys was lower than eGFRcr in Japanese hospital-based patients with COPD^8^. The current study further revealed that eGFRcys was lower than eGFRcr in the subjects with AFL among the community-based population. In addition the value of c-statistics for mortality was significantly higher in Cys-based eGFR than Cr-based eGFR. Accordingly, eGFRcys might be more suitable than eGFRcr for evaluating renal function in subjects with AFL.

Regarding the rate of overlap of AFL and CKD, previous hospital-based studies described that the prevalence of CKD was higher in patients with COPD than in those without COPD and that patients with COPD are 1.6–6.3 times more likely to develop CKD than those without COPD^12–14^. Our study further revealed that the more severe the AFL is, the more likely is the patient to have CKD, and vice versa, in the community-based population. These findings indicate that AFL and CKD are strongly associated and that these two diseases frequently overlap.

The strong association between AFL and CKD overlap and mortality has been previously reported in some specific situations, such as in patients with CKD after vascular surgery^15^, in patients with advanced CKD^16,17^, and in patients with COPD after discharge^18^. Our findings further revealed the effect of the overlap of AFL and CKD on mortality in the community-based population. However, another study reported a different result, which showed that restrictive lung dysfunction, but not obstructive lung impairment, had an effect on life prognosis in CKD patients^19^. One of the possible reasons for this inconsistency might be the use of serum Cr-based eGFR for evaluating the presence of CKD in previous studies because serum Cr-based eGFR might overestimate renal function in subjects with COPD. Further studies are required to confirm the usefulness of serum cystatin-based eGFR in the early detection of CKD.

Although the mechanism of how COPD and CKD are linked to each other is unknown, the involvement of systemic inflammation might be one of the possible explanations. Increased inflammatory mediators, such as tumour necrosis factor-α, interleukin-6, and C-reactive protein, are detected in the blood of patients with COPD^20^ and CKD^21^. Such systemic inflammatory factors may induce the development and progression of COPD and CKD. Furthermore, it is speculated that systemic inflammation is involved in the development of cardiovascular diseases^21^, and the various inflammatory mediators derived from lung or kidney damage might induce multiorgan dysfunction.

This study had several notable limitations. First, in clinical practice, COPD is diagnosed as FEV1/FVC < 70% on spirometry after the inhalation of bronchodilators. However, in epidemiological studies, a bronchodilator is not usually used in health check-up participants who are not suspected of having COPD. Therefore it is not possible to know true prevalence of COPD in this study population. However, a previous Japanese study showed that the prevalence of airflow limitation diagnosed by spirometry and COPD confirmed by further examination was 11.3% and 8.4%, respectively, in community-based population, indicating the majority (74.3%) of airflow limitation was confirmed as COPD^22^. It also showed that COPD was observed more in men and smokers, than in women and never-smokers. Second, this study had no information on thyroid function and the use of immunosuppressive agents that might affect CysC levels^11^. However, it is assumed that the number of such subjects was very small because the study subjects were generally healthy check-up participants. Third, the presence of CKD and AFL was evaluated only at baseline, and the changes of these conditions were not assessed after the baseline survey. Fourth, the questionnaire used in this study did not contain the questions on socioeconomic status and educational level that are associated with mortality. Therefore, we could not adjust the confounding effect of these factors. Fifth, the number of the subjects enrolled in this analysis was less than half of the screening attendee. Although the effect of selection bias seems to be limited, the caution is required to generalize the study results.

In conclusion, this study showed that AFL and CysC-based CKD are strongly associated and that their overlap is a significant risk factor for mortality in community-based populations. To reduce the burden with respect to prognosis and medical cost, further studies are warranted to clarify the significance of the early detection of these diseases.

## Methods

### Study subjects

The subjects of this cohort study were participants of community-based annual health check-ups in the town of Takahata, Japan from 2004 to 2006 who agreed to be included in this study and who were followed up until the end of 2013. Among 3,523 subjects who agreed to participate in this study, 2,290 were excluded because of missing essential data such as serum Cr level, serum CysC level, urine test results, and pulmonary function test results. Consequently, a total of 1,233 subjects were included in the final analysis. The baseline characteristics of the study subjects and the excluded subjects showed no significant difference, except the slightly higher age and the lower prevalence of hyperlipidaemia in the excluded subjects than the study subjects (Supplementary Table 1). This study was approved by the Ethical Review Committee of Yamagata University Faculty of Medicine (Yamagata University, April 2006, No. 1). Informed consent was obtained from all patients, and the procedures were conducted in accordance with the Declaration of Helsinki. The details of the study design, recruitment procedures, and profile of the study participants have been described previously^23^.

### Measurements

At the baseline survey, we collected laboratory test data and medical history information obtained using an interview sheet. Systolic and diastolic blood pressures were measured using a mercury sphygmomanometer, with the subject in a sitting position after resting for >5 min. Hypertension was defined as systolic blood pressure ≥140 mmHg or diastolic blood pressure ≥90 mmHg or the use of antihypertensive drugs. Diabetes was defined as fasting blood glucose level ≥126 mg/dL, haemoglobin A1c ≥ 6.5%, or the use of hypoglycaemic drugs. Hyperlipidaemia was defined as serum total cholesterol level ≥220 mg/dL or the use of lipid-lowering drugs. Subjects who reported that they drank alcohol daily or occasionally were classified as having alcohol consumption. Current smoking was defined as having a smoking habit. Generally, COPD is defined as the ratio of forced expiratory volume in 1 s to forced vital capacity (FEV1*/*FVC) < 70% on spirometry performed after the inhalation of bronchodilators^24^; however, it is difficult to use bronchodilators in community-based health check-ups. Therefore, in the current study, we used the presence of airflow limitation, defined as FEV1% (FEV1/FVC) < 70% without bronchodilator use. The severity of airflow limitation was classified according to the Global Initiative for Chronic Obstructive Lung Disease criteria using FEV1% (% predicted) as follows: ≥80%, grade I; 50–79%, grade II; 30–49%, grade III; and <30%, grade IV^3^. CKD was defined as eGFR <60 mL/min/1.73 m^2^ or dipstick proteinuria ≥1+ in accordance with the definition of the Japanese Society of Nephrology^25^. eGFRcr and eGFRcys were calculated using serum Cr or CysC by the Japanese equation^11^. CKDcr was defined as eGFRcr <60 mL/min/1.73 m^2^ or dipstick proteinuria ≥1 + , and CKDcys was defined as eGFRcys <60 mL/min/1.73 m^2^ or dipstick proteinuria ≥1 + .

### Outcome

We followed up the study subjects until 2013 (median 9.3 years) and collected information on death and cause of death from death certificates.

### Statistical analysis

Data are presented as median ± standard deviation unless otherwise indicated. For comparisons of continuous values and prevalence, we used the t-test and the chi-square test. To examine the correlation between the prevalence of CKD and the severity of airflow limitation, we used the Cochran-Armitage trend test. To evaluate the goodness of fit in a logistic regression model for mortality using eGFRcr or eGFRcys, we performed a c-statistics analysis. To investigate the association among CKD, AFL, and all-cause death, we performed Kaplan-Meier analysis with log-rank test and Cox proportional hazard model analysis adjusted for age, sex, smoking, alcohol consumption, body mass index, hypertension, diabetes, and hyperlipidaemia. We performed all statistical analyses using JMP version 10 (SAS Institute Inc., Cary, NC, USA) and EZR software version 1.41^26^. A P-value <0.05 was considered statistically significant.

## Supplementary information

Supplementary information.

## Data Availability

The dataset analysed in this study are not available owing to ethical reasons.
