# Supplementary material for: Association among chronic kidney disease, airflow limitation, and mortality in a community-based population: The Yamagata (Takahata) study
Source: Sci Rep. 2020 Mar 27;10:5570. doi: 10.1038/s41598-020-62540-8 (PMC7101320; doi:10.1038/s41598-020-62540-8)
Supplement: Supplementary file 1 — Supplementary information. [file 41598_2020_62540_MOESM1_ESM.docx]

Supplementary Information

**Title:**

Association among chronic kidney disease, airflow limitation, and mortality in a community-based population: The Yamagata (Takahata) study

**Authors:**

Natsuko Suzuki, Eri Matsuki, Akira Araumi, Sakiko Ashitomi, Sayumi Watanabe, Kosuke Kudo, Kazunobu Ichikawa, Sumito Inoue, Masafumi Watanabe, Yoshiyuki Ueno, Kenichi Ishizawa, Takamasa Kayama, Tsuneo Konta

Supplementary table 1. Baseline characteristics of the study subjects and the subjects excluded from the analysis.

|  | Study subjects  N = 1,233 | Excluded subjects  N = 2,290 | P-value |
| --- | --- | --- | --- |
| Age (years) | 63.7 ± 9.8 | 62.0 ± 10.7 | <0.01 |
| Male sex (%) | 569 (46.7%) | 1,015 (44.3%) | 0.30 |
| Smoking status (%) | 164 (13.3%) | 293 (12.8%) | 0.08 |
| Alcohol consumption (%) | 516 (41.8%) | 951 (41.5%) | 0.85 |
| Body mass index (kg/m^2^) | 23.5 ± 3.2 | 23.5 ± 3.2 | 0.79 |
| Serum Cr (mg/dL) | 0.67 ± 0.16 | 0.68 ± 0.24 | 0.59 |
| Serum cystatin C (mg/L) | 0.95 ± 0.19 | 0.95 ± 0.24 | 0.83 |
| Hypertension (%) | 532 (43.2%) | 1,019 (45.1%) | 0.26 |
| Diabetes (%) | 67 (5.8%) | 113 (5.3%) | 0.62 |
| Hyperlipidaemia (%) | 378 (30.7%) | 796 (35.2%) | 0.01 |

Cr, creatinine
